# Allocation and characterisation factors for absolute environmental sustainability assessment

**DOI:** 10.1038/s41597-026-07797-w

**Published:** 2026-07-04

**Authors:** Jan Marcus Hartmann, David Yang Shu, André Bardow, Niklas von der Assen

**Affiliations:** 1 Institute of Technical Thermodynamics, RWTH Aachen University, 52062 Aachen, Germany; 2 Energy and Process Systems Engineering, Department of Mechanical and Process Engineering, ETH Zurich, Zurich, 8092 Switzerland

## Abstract

Absolute environmental sustainability assessment (AESA) enables the comparison of the environmental burdens from economic sectors against sector-specific thresholds for environmental sustainability. Implementing AESA requires data to establish sector-specific sustainability thresholds and to characterise the environmental burden of elementary flows that are exchanged between the sector and the environment. Yet, a reusable dataset for AESA is currently lacking. The following dataset comprises the necessary data to perform a planetary-boundary-based AESA. The dataset includes allocation factors for 163 economic sectors across 44 countries and 5 Rest-of-World regions to determine environmental sustainability thresholds. The allocation factors are calculated using four allocation principles that combine population-based allocation with allocation based on direct or total, and on consumption- or production-based value generated by the economic sector. Additionally, the dataset provides characterisation factors for the 2684 elementary flows within standard and prospective ecoinvent v3.10.1 databases, enabling the assessment of the elementary flows’ environmental burden across all global planetary boundary categories except novel entities.

## Background & Summary

Anthropogenic systems, such as economic sectors, must be transformed to achieve environmental sustainability and reverse environmental pressures^1^. To guide the transformation process, system configurations need to be assessed in terms of environmental burden through absolute environmental sustainability assessment (AESA)^2,3^. In AESA, the system’s environmental burdens are compared against system-specific thresholds for environmental sustainability^3^.

System-specific thresholds can be derived from the planetary boundary framework^1^ which defines the *safe operating space for humanity* (SOS) as the sustainable limit to all anthropogenic environmental burden. Anthropogenic systems operating within their allocated share of the SOS avoid irreversible environmental change and are thus sustainable^1^. To this end, when assessing economic sectors, a share of the SOS has to be allocated to the economic sector under study to obtain sector-specific environmental sustainability thresholds. Current literature suggests allocation principles^2–7^ and provides allocation factors for selected economic sectors in selected countries or regions^8–11^. However, no dataset exists that provides allocation factors for all formal economic sectors in multiple countries or world regions. Additionally, literature lacks a transparent and reusable method to generate temporally updated allocation factors, which is particularly necessary as economic sectors evolve over time.

In planetary-boundary-based AESA, anthropogenic systems are considered sustainable if their environmental burden remains below the system-specific sustainability threshold. The system’s environmental burden is commonly determined via life cycle assessment, a standardised, holistic method to assess multiple environmental burdens of an anthropogenic system along its entire life cycle^12^. To perform a life cycle assessment, all mass and energy flows that are exchanged between the anthropogenic system and the environment, the so-called elementary flows, to fulfil the functional unit need to be modelled^12^. In AESA, the practitioner must ensure that the selected functional unit and included processes during life cycle assessment are consistent with those applied during allocation^3^. To model elementary flows and facilitate data acquisition, life cycle assessment practitioners use databases such as ecoinvent^13^. Afterwards, the elementary flows’ burden is calculated across environmental burden categories by multiplying the elementary flows with characterisation factors that link the flow and the burden category^12^.

Characterisation factors for planetary-boundary-based AESA can be distinguished into two categories: 1) characterisation factors that link elementary flows to the environmental burden categories of the Environmental Footprint method, for which environmental burdens are compared against an adapted SOS; and 2) characterisation factors that link elementary flows directly to the planetary boundary categories, for which environmental burdens are compared directly against the SOS^14^. For the latter set of characterisation factors, current literature only provides characterisation factors for all elementary flows within the standard ecoinvent v3.5 database (2018)^8,15^. For the land-system change category, an additional challenge arises: When applying characterisation factors from current literature^8,15^ that use a time-dependent functional unit—i.e., output of the anthropogenic system per unit of time—the resulting burden is also time-dependent. Time-dependent burdens arise because the characterisation factors address elementary flows related to land transformation^8,15^. In contrast, the SOS for land-system change is defined independently of time^1^. Consequently, comparing the time-dependent burden on land-system change with the time-independent SOS for land-system change requires aggregating the burden over the entire time horizon of the anthropogenic system’s operation. The choice of the time horizon introduces subjectivity and uncertainty. Doka *et al*.^16^ suggest redefining the SOS as land that is available for anthropogenic occupation, and characterising the elementary flows of land occupation, thus circumventing the subjective choice of a time horizon. However, characterisation factors corresponding to this definition of the SOS are currently lacking.

To address these gaps, we provide a dataset of allocation factors for 163 economic sectors, as defined by the EU’s NACE Rev.1 classification^17^, in 44 countries and 5 Rest-of-World regions. The allocation factors are calculated using four allocation principles that combine population-based allocation with economic value-based allocation. Economic value-based allocation is proportional to either direct or total consumption- or production-based value generated by the economic sector. We focus on population-based and economic value-based allocation principles, as these principles are widely applied by AESA practitioners and the data required to operationalise these principles is commonly available. However, we acknowledge that many allocation principles exist^4^, and that the subjective choice of an allocation principle can have a substantial impact on the resulting allocation factors^18^. To address this issue, we introduce a systematic and transparent method for calculating allocation factors.

Furthermore, we provide an updated dataset of characterisation factors, linking all 2684 elementary flows included in the standard and prospective ecoinvent v3.10.1 (2025) databases^13,19^ directly with the global planetary boundary categories for climate change, ocean acidification, change in biosphere integrity, stratospheric ozone depletion, the phosphorus cycle, the nitrogen cycle, freshwater use and atmospheric aerosol loading, as defined by Steffen *et al*.^1^. In addition, we characterise the burdens of the elementary flows across the planetary boundary category for land-system change based on land available for anthropogenic occupation, as defined by Doka *et al*.^16^.

## Methods

### Allocation factors

We determine the allocation factors for economic sectors across geographical scopes (e.g. countries or regions), based on theories of distributive justice. Theories of distributive justice postulate that a just allocation is always human-centric^4^. Although economic sectors are not human entities, they create value for humans. Hence, we determine allocation factors with the two-step approach proposed by Hjalsted *et al*.^4^: First, we allocate a share to the geographical scope, based on the share of global population living within the geographical scope. This allocates an equal share to each individual human, following the distributive justice theory of Egalitarianism^4^. Second, we allocate a share of the geographical scope’s allocated share to each economic sector within the geographical scope, based on the value that the economic sector generates for humans living in the geographical scope relative to the value generated by all sectors within the geographical scope, following the distributive justice theory of Utilitarianism^4^.

An economic sector generates direct and indirect value from both consumption and production perspectives. From the consumption perspective, direct value is generated when an economic sector’s output is directly consumed by the final consumer – such as individuals, households, and governmental institutions, who gain utility from the output. Indirect value is generated when the sector’s output is used by the sector’s downstream supply chain to produce output, which in turn is consumed by the final consumer. The sector’s downstream supply chain comprises other economic sectors in the same or other geographical scopes. The sum of direct and indirect consumption-based value is the total consumption-based value generated by an economic sector. We approximate the direct and total consumption-based value generated by an economic sector using the economic indicator of *final consumption expenditure (FCE)*, defined as the monetary expenditure on goods and services for final use by individuals, households, governments, and non-profit institutions.

From the production perspective, direct value is generated within the sector, for example, by paying wages to its employees. Indirect value is generated as the sector’s input is produced by the sector’s upstream supply chain, which generates value within itself, for example, by paying wages to their employees. The sector’s upstream supply chain comprises other economic sectors in the same or other geographical scopes. The sum of direct and indirect production-based value is the total production-based value generated by an economic sector. We approximate the direct and total production-based value generated by an economic sector using the economic indicator of *gross value added (GVA)*, which represents the monetary value of goods and services produced by the sector minus the monetary value of intermediate inputs used in production.

#### Mathematical formulation of allocation principles

We define four allocation principles to determine the allocation factor for a sector-scope pair $({j}^{* },{s}^{* })$, where $j$* is an economic sectors from the set of sectors ${\mathscr{J}}$ and $s$* is a geographical scope from the set of geographical scopes ${\mathscr{S}}$. The set of sectors ${\mathscr{J}}$ and geographical scopes ${\mathscr{S}}$ are determined by an input-output model of the global economy that serves as the data source to which the allocation principles are applied.

We define the allocation principles following the two-step approach by Hjalsted *et al*.^4^. Accordingly, we obtain the allocation factors ${SoSO}{S}_{j* ,s* }^{p,q}$ for the sector-scope pair $({j}^{* },{s}^{* })$ and for the four allocation principles differing between consumption and production value perspectives, $p\in \left\{{\rm{cons}},\,{\rm{prod}}\right\}$, and direct and total value perspectives, $q\in \left\{{\rm{direct}},{\rm{total}}\right\}$:

1 $${SoSO}{S}_{j* ,s* }^{p,q}=\sum _{r\epsilon {\mathscr{S}}}{SoSO}{S}_{r}\cdot {SoSO}{S}_{j* ,s* ,\,r}^{p,q}$$

In Eq. 1, ${SoSO}{S}_{r}$ is the share of global population living in a geographical scope $r$ (cf. Eq. 2) and ${{SoSOS}}_{j* ,s* ,r}^{p,{q}}$ is the share of value generated within $r$ by the sector-scope pair $({j}^{* },{s}^{* })$ (cf. Eqs. 3 and 4).

2 $${SoSO}{S}_{r}=\frac{{PO}{P}_{r}}{{PO}{P}^{{\rm{global}}}}$$

In Eq. 2, ${PO}{P}_{r}$ denotes the population of geographical scope $r$ and ${PO}{P}^{{\rm{global}}}$ the global population.

The value-share ${{SoSOS}}_{j* ,s* ,r}^{p,{q}}$ is determined for the four allocation principles. Consequently, the value-share ${{SoSOS}}_{j* ,s* ,r}^{p,{q}}\,$ corresponds to the share of final consumption expenditure or gross value added in $r$, for consumption or production-based perspectives, respectively, that is directly, or directly and indirectly (total) generated by $({j}^{* },{s}^{* })$ (cf. Eqs. 3, 4).

3 $${{SoSOS}}_{j* ,s* ,r}^{{\rm{cons}},q}=\frac{{{FCE}}_{j* ,s* ,r}^{q}}{{{FCE}}_{r}},\,q\in \left\{{\rm{direct}},{\rm{total}}\right\}$$

4 $${{SoSOS}}_{j* ,s* ,r}^{{prod},\,q}=\frac{{{GVA}}_{j* ,s* ,r}^{q}}{{GV}{A}_{r}},\,q\in \left\{{\rm{direct}},{\rm{total}}\right\}$$

In Eq. 3, ${{FCE}}_{j* ,s* ,r}^{q}$ is the final consumption expenditure generated by $({j}^{* },{s}^{* })$ in $r$ considering direct and total value generated $q\in \left\{{\rm{direct}},{\rm{total}}\right\}$, and ${{FCE}}_{r}$ is the sum of direct final consumption expenditure in $r$ across all sector-scope pairs $(j,s)$, where $j\in {\mathscr{J}}$ and $s\in {\mathscr{S}}$. In Eq. 4, ${{GVA}}_{j* ,s* ,r}^{q}$ is the gross value added generated by $({j}^{* },{s}^{* })$ in $r$ considering direct and total value generated $q\in \left\{{\rm{direct}},{\rm{total}}\right\}$, and ${{GVA}}_{r}$ is the sum of direct gross value added in $r$ across all sector-scope pairs $(j,s)$. Direct final consumption expenditure and direct gross value added can be directly obtained from the input-output model.

The total final consumption expenditure and total gross value added are obtained by scaling the direct final consumption expenditure and direct gross value added, respectively (cf. Eqs. 5, 6).

5 $${{FCE}}_{j* ,s* ,r}^{{\rm{total}}}={s}_{j* ,s* ,r}^{{\rm{FCE}}}\cdot {{FCE}}_{j* ,s* ,r}^{{\rm{direct}}}$$

6 $${{GVA}}_{j* ,s* ,r}^{{\rm{total}}}={s}_{j* ,s* ,r}^{{\rm{GVA}}}\cdot {{GVA}}_{j* ,s* }^{{\rm{direct}}}$$

In Eq. 5, the scaling factor ${s}_{j* ,s* ,r}^{{\rm{FCE}}}$ captures the consumption-based value generation of the downstream supply chain that is induced by the sector-scope pair’s $({j}^{* },{s}^{* })$ output. The scaling factor is calculated following the method of Oosterhoff *et al*.^6^, which is omitted here for brevity. In Eq. 6, the scaling factor ${s}_{j* ,s* ,r}^{{\rm{GVA}}}$ captures the production-based value generation of the upstream supply chain that is induced by the sector-scope pair’s $({j}^{* },{s}^{* })$ input.

7 $${s}_{j* ,s* ,r}^{{\rm{GVA}}}={\alpha }_{r,j* ,s* }\cdot {m}_{j* ,s* }({{GVA}}_{j* ,s* }^{{\rm{direct}}})$$

In Eq. 7, ${m}_{j* ,s* }\left({{GVA}}_{j* ,s* }^{{\rm{direct}}}\right)$ is the type I gross-value-added multiplier for each $({j}^{* },{s}^{* })$, as proposed by Keček *et al*.^20^. The type I gross-value-added multiplier captures the production-based value generation of the upstream supply chain that is induced by the sector-scope pair’s $({j}^{* },{s}^{* })\,$ input, without disaggregating by the geographical scope $r$. In Eq. 7, ${\alpha }_{r,j* ,s* }$ is the share of inputs into $({j}^{* },{s}^{* })\,$ from geographical scope $r$.

8 $${\alpha }_{r,j* ,s* }\,=\,\sum _{i}{\varphi }_{i,r,j* ,s* }$$

In Eq. 8, ${\varphi }_{i,r,j* ,s* }$ is the share of inputs into $({j}^{* },{s}^{* })\,$ from each sector-scope pair $(i,r)$.

9 $${\varphi }_{i,r,j* ,s* }=\,\left\{\begin{array}{c}\begin{array}{cc}\frac{{z}_{i,r,j* ,s* }}{{T}_{j* ,s* }} & \,{if}\,(i,r)\ne ({j}^{* },{s}^{* })\end{array}\\ \begin{array}{cc}\frac{{z}_{i,r,j* ,s* }+{{GVA}}_{j* ,s* }^{{\rm{direct}}}}{{T}_{j* ,s* }} & {if}\,(i,r)=({j}^{* },{s}^{* })\end{array}\end{array}\right.$$

In Eq. 9, ${z}_{i,r,j* ,s* }$ is an input from the sector $i{\mathscr{\in }}{\mathscr{J}}$ in geographical scope $r\,\epsilon \,{\mathscr{S}}$, ${{GVA}}_{j* ,s* }^{{\rm{direct}}}$ is the direct gross value added of $({j}^{* },{s}^{* })$, and ${T}_{j* ,s* }$ is the total input into the sector-scope pair $({j}^{* },{s}^{* })$, which is calculated with Eq. 10.

10 $${T}_{j* ,s* }=\,\left(\sum _{(i,r)}{z}_{i,r,j* ,s* }\right)+{{GVA}}_{j* ,s* }^{{\rm{direct}}}$$

Since the total consumption-based and production-based values of a sector include the downstream and upstream supply chains, respectively, the same economic value can be attributed to multiple economic sectors. Consequently, allocation factors are not additive across sectors, and their sum generally exceeds one. As a result, aggregating allocation factors—such as when accounting for the same sector across multiple geographic scopes—can lead to double counting of consumption-based or production-based values.

This limitation can be illustrated using a simplified economy consisting of only one geographical scope and of two economic sectors A and B, where sector A supplies sector B. The total consumption-based value of sector A consists of its own direct consumption-based value plus the share of sector B’s consumption-based value that is attributable to inputs from sector A. In contrast, the total consumption-based value of sector B equals only its direct consumption-based value because it does not supply any downstream sector.

If the total consumption-based values of sectors A and B are summed, the resulting value exceeds the economy’s overall consumption-based value, which is simply the sum of the sectors’ direct consumption-based values. The excess arises because part of sector B’s value is counted twice: once as part of sector B’s own total value and again as part of sector A’s total value. An analogous overlap occurs for production-based values, where upstream supply chain relationships lead to double counting.

Consequently, the sum of value-shares ${{SoSOS}}_{j* ,s* ,r}^{p,{q}}$ exceeds 1, when total value generation is considered, as the same value is attributed to multiple economic sectors. For the same reason, the sum of allocation factor ${SoSO}{S}_{j* ,s* }^{p,q}$ resulting from the value-share also exceeds 1.

#### Application of allocation principles

We obtain all data required for calculating consumption-based and production-based values from the multi-regional input-output database, EXIOBASE 3.9.6^21^, which is an input-output model of the global economy, and by using the most recent industry-by-industry database for 2022^21^. Therefore, we determine the allocation factors for 163 economic sectors across 44 countries and 5 Rest-of-World regions, i.e., 49 geographical scopes, as included in EXIOBASE 3^22^. For population data, we use country-specific figures provided by the World Bank for 2022^23^ for all countries that are explicitly defined in EXIOBASE 3^22^. We align the Rest-of-World regions in EXIOBASE 3 with corresponding World Bank regional classifications^24^ (cf. Table 1). For each Rest-of-World region, we include the population data of all countries that are not explicitly defined in EXIOBASE 3^22^.

**Table 1:** Mapping the Rest-of-World regions in EXIOBASE 3 with corresponding World Bank regional classifications.

Rest-of-World regions in EXIOBASE 3	World Bank regional classification
RoW Asia and Pacific	East Asia & Pacific South Asia
RoW America	Latin America & Caribbean North America
RoW Europe	Europe & Central Asia
RoW Africa	Sub-Saharan Africa
RoW Middle East	Middle East & North Africa

### Characterisation factors

Our calculation of the characterisation factors primarily builds on the work of Ryberg *et al*.^15^ and Bachmann *et al*.^8^ Ryberg *et al*. developed characterisation methods to obtain characterisation factors for planetary-boundary-based life cycle impact assessment, while Bachmann *et al*.^8^ adapted these characterisation methods and published characterisation factors for all 2080 elementary flows included in ecoinvent v3.5^13^. In this work, we further adapt the characterisation method for land-system change and apply the resulting characterisation methods to all 2684 elementary flows within standard and prospective ecoinvent v3.10.1.

In ecoinvent, elementary flows are distinguished by the type of mass or energy emitted or extracted, the main compartment of emission (i.e. air, water or soil) or extraction (i.e. natural resources), and additional sub-compartments. The standard ecoinvent v3.10.1 database^13^ and the prospective ecoinvent v3.10.1 databases, generated using premise 2.1.9^19^, include 2648 and 2684 elementary flows, respectively. The increase in elementary flows between v3.5 and v3.10.1 is due to additional masses, energies, compartments and sub-compartments.

Furthermore, v3.10.1 includes changes to the name, compartment and sub-compartment of elementary flows compared to v3.5. When changes between v3.5 and v3.10.1 involve only synonyms, new or renamed compartments, or sub-compartments, we transfer the original characterisation factors from v3.5 to the corresponding updated elementary flows in v3.10.1. To address flows newly added or previously unaccounted for in v3.5, we introduce characterisation factors for the following planetary boundary categories: climate change, biosphere integrity, ocean acidification, stratospheric ozone depletion, and atmospheric aerosol loading. We introduce new characterisation factors for land-system change by characterising land occupation. To account for the burden on the nitrogen cycle, we introduce a new elementary flow and characterisation factor. For the planetary boundaries on climate change and biosphere integrity—each of which is defined by two control variables—the dataset includes characterisation factors for only one control variable per boundary. Additionally, regionalized burden categories included in the planetary boundary framework are not represented in the dataset.

#### Climate change, change in biosphere integrity and ocean acidification

For the planetary boundary categories, climate change, change in biosphere integrity and ocean acidification, we add characterisation factors for *‘Carbon dioxide, to soil or biomass stock’* to the compartment *‘soil’* by copying and negating the characterisation factors for *‘Carbon dioxide, fossil’* to *‘air’*, in accordance with other life cycle impact assessment methods, such as the Environmental Footprint 3.1^25^ Furthermore, we assign a characterisation factor for *‘Ketene’* to *‘air’* by copying the factor from *‘NMVOC, non-methane volatile organic compounds’* to *‘air’*, as *‘Ketene’* does not have a specified carbon content.

Bachmann *et al*.^8^ calculate characterisation factors for the burden of land occupation on change in biosphere integrity using the method provided by Galán-Martín *et al*.^26^, which uses data for mean species abundance loss from Hanafiah *et al*.^27^. However, Hanafiah *et al*. do not provide data for all types of land occupation present in the v3.10.1 database. Hence, we map the types of land occupation introduced between v3.5 and v3.10.1 to the classifications used by Hanafiah *et al*., as detailed in Table 2. If multiple values are provided by Hanafiah *et al*. for a given type of land occupation, we use the maximum values as a conservative approximation.

**Table 2:** Mapping the classification of land occupation in ecoinvent v3.10.1 to the classification of land occupation in Hanafiah *et al*. and their respective values for loss in mean species abundance.

Classification in ecoinvent v3.10.1	Classification in Hanafiah *et al*.	Loss in mean species abundance
Occupation, annual crop, greenhouse	Permanent crop, intensive	0.9
Occupation, forest, unspecified	Forest, intensive, short-cycle	0.8
Occupation, permanent crop, non-irrigated, intensive	Permanent crop, intensive	0.9
Occupation, seabed, unspecified	Sea and ocean	0
Occupation, urban, continuously built	Urban, continuously built	0.95
Occupation, urban, green area	Urban, discontinuously built	0.95

#### Stratospheric ozone depletion

We characterise *‘Dichloromethane’* to *‘air’* using the characterisation method provided by Ryberg *et al*.^15^, assuming an atmospheric lifetime of dichloromethane of 0.5 years^28^ and a fractional release ratio of 1.

#### Atmospheric aerosol loading

We characterise the elementary flow *‘Carbon’* to *‘air’* with the characterisation factor for *‘Carbon-14’* to *‘air’*. Furthermore, we assign a characterisation factor to *‘Ketene’* to *‘air’* by copying the factor from *‘NMVOC, non-methane volatile organic compounds’* to *‘air’* and the sub-compartment *‘unspecified’*, as *‘Ketene’* does not have a specified carbon content.

#### Nitrogen cycle

Nitrogen application to soil via fertilisers impacts the planetary boundary category for the nitrogen cycle^1^. However, nitrogen application to soil via fertilisers is not included as an elementary flow in ecoinvent, but as a technosphere flow^13,29^. To assess the burden on the nitrogen cycle, we introduce the new elementary flow *‘N-supply’*, emitted to the compartment *‘soil’*. This elementary flow is added to all processes that supply nitrogen application via fertilizers as a technosphere flow. Specifically, we consider all processes in ecoinvent v3.10.1^13^ that include the term “nutrient supply” in their process name and provide either *‘organic nitrogen fertiliser, as N’* or *‘inorganic nitrogen fertiliser, as N’* as the technosphere flow. Hence, if a process supplies 1 kg of nitrogen to an agricultural system, 1 kg of the *‘N-supply’* elementary flow is emitted to the compartment *‘soil’*. We characterise the elementary flow *‘N-supply’* to the compartment *‘soil’* with the factor 1e-9 to convert from kilograms to teragrams.

#### Land-system change

We provide a novel set of characterisation factors for land-system change to enable the comparison of the environmental burden on land-system change with the SOS defined by Doka *et al*.^16^. We characterise all elementary flows representing anthropogenic land occupation in ecoinvent v3.10.1 with the factor 1e-12, converting from square meters to million square kilometres, except the elementary flows beginning with *‘Occupation, forest’*, *‘Occupation, grassland, natural’*, and *‘Occupation, seabed’*, as well as the elementary flows *‘Occupation, unspecified, natural (non-use)’* and *‘Occupation, shrub land, sclerophyllous’*. We exclude these elementary flows since the SOS defined by Doka *et al*.^16^ corresponds to the land that is available for anthropogenic occupation. Land that is available for anthropogenic occupation is calculated as the total area of ice-free land surface minus the minimum area that should be covered by forest^16^. The minimum area that should be covered by forest is retrieved from Steffen *et al*.^1^. Hence, we do not characterise *‘Occupation, forest’*, conservatively assuming that it reflects existing forest cover rather than anthropogenic forest cover expansion. We do not characterise *‘Occupation, grassland, natural’*, *‘Occupation, unspecified, natural (non-use)’* and *‘Occupation, shrub land, sclerophyllous’*, assuming that no forest cover has been removed from these natural land-systems by anthropogenic activity. We further do not characterise *‘Occupation, seabed’* because it does not involve the occupation of terrestrial land area.

## Data Records

### Allocation factors

The dataset is openly accessible in the Zenodo repository *Allocation Factors for Absolute Environmental Sustainability Assessment*^30^, which includes the Excel file *Allocation Factors.xlsx* that provides the allocation factors for 163 economic sectors, across 44 countries and 5 Rest-of-World regions, for four allocation principles (cf. Section 2.1). The Excel file consists of one sheet, with the first column of the sheet containing country abbreviations, based on the ISO 3166-1 alpha-2 standard^31^, along with abbreviations for the Rest-of-World (RoW) regions for Asia and Pacific (**WA)**, America (**WL)**, Europe (**WE)**, Africa (**WF)**, and the Middle East (**WM)**. The second column includes the economic sector names, as defined by the EU’s NACE Rev.1 classification^17^. The third and fourth column include the allocation factors calculated based on the sector’s direct and total consumption-based value for direct and total final consumption expenditure, respectively. The fifth and sixth column include the allocation factors calculated based on the sector’s direct and total production-based value for direct and total gross value added, respectively.

### Characterisation factors

The dataset is openly accessible in the Zenodo repository *Characterisation Factors for Planetary-Boundary-Based Absolute Environmental Sustainability Assessment*^32^, which includes the following data for the calculation of an anthropogenic system’s (e.g. economic sector’s) environmental burden across the planetary boundary categories.

The Excel file *Characterisation factors_for_ecoinvent3101.xslx*, which includes the characterisation factors linking the 2684 elementary flows of the standard and prospective ecoinvent v3.10.1 databases^13,19^ to all global planetary boundary categories except novel entities^1^. The Excel file contains two sheets, with the first including the characterisation factors, and the second the values for the global safe operating space.

In the first sheet, the first five columns include identifiers for the elementary flow within the ecoinvent v3.10.1 databases. Specifically, the columns include from left to right: 1) the unique code identifying each elementary flow in the ecoinvent databases, 2) the name of the elementary flow, 3) the environmental compartment to which the elementary flow is emitted or from which it is extracted, 4) the respective sub-compartment, and 5) the unit of the elementary flow. The next eight columns include the characterisation factors for the following planetary boundary categories, listed from left to right: climate change, change in biosphere integrity, ocean acidification, stratospheric ozone depletion, phosphorus cycle, nitrogen cycle, freshwater use, atmospheric aerosol loading and land-system change. The second row of the first sheet includes the control variables and their respective units associated with each planetary boundary category.

In the second sheet, the first column includes the name of the planetary boundary category, the second column includes the unit of the safe operating space and the third column the value of the safe operating space for the planetary boundary category.

## Technical Validation

We calculate the allocation factors (cf. Section 2.1), based on data from EXIOBASE 3.9.6^21^ and the World Bank^23^ as provided and verify the mathematical implementation of our calculation through consistency checks. Specifically, we ensure that allocation factors computed from direct consumption- or production-based value sum to one.

The characterisation factors provided in this dataset build on existing characterisation methods, primarily from Bachmann *et al*.^8^, for all planetary boundary categories except land-system change. Thus, for validation of the characterisation factors, we perform a life cycle assessment for all processes in the ecoinvent 3.10.1 database using 1) the characterisation factors of Bachmann *et al*. and 2) the characterisation factors provided in this dataset, for all planetary boundary categories except for land-system change and for the nitrogen cycle. As our characterisation model for land-system change is based on land-occupation and thus differs from Bachmann *et al*., we ensure that the land-system change burden corresponds to the land-occupation of processes in ecoinvent 3.10.1, with the exclusions described in Section 2.2.5. We exclude the nitrogen cycle, as the characterisation factors and characterised elementary factor are identical between Bachmann *et al*. and the provided dataset.

Although developed for the elementary flows in ecoinvent 3.5, the characterisation factors by Bachmann *et al*. cover many elementary flows included in ecoinvent 3.10.1. We compare the deviation of the environmental burdens calculated with the characterisation factors provided in this dataset against the environmental burdens calculated with the characterisation factors from Bachmann *et al*. for each process in ecoinvent 3.10.1 and for each considered planetary boundary category.

**Fig. 1:**
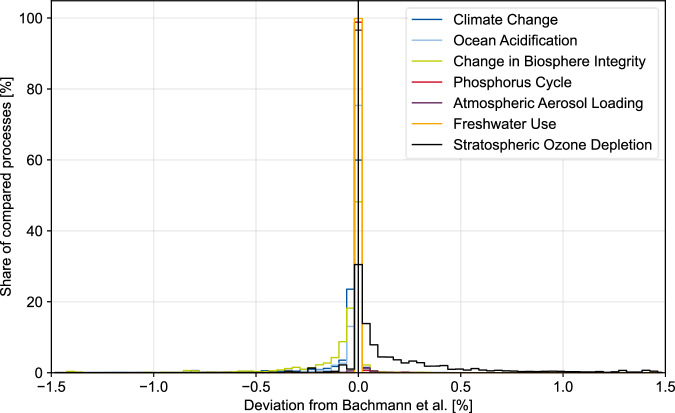
Distribution of percentage deviations between environmental burdens calculated with the characterisation factors provided in this dataset and the characterisation factors from Bachmann *et al*. for all processes in ecoinvent 3.10.1.

The environmental burdens of most ecoinvent 3.10.1 processes deviate by less than 1%, when using the characterisation factors provided in this dataset instead of those of Bachmann *et al*. For climate change, ocean acidification, change in biosphere integrity, phosphorus cycle, atmospheric aerosol loading, and freshwater use, 97.1%, 97.7%, 94.0%, 99.8%, 99.8%, and 99.8% of all processes, respectively, show deviations below 1% (cf. Figure 1). For stratospheric ozone depletion, 86.9% of processes show a deviation below 1%, and 96.9% fall below 5% (cf. Figure 1). Overall, deviations in environmental burden compared to Bachmann *et al*. are small and reflect the additional characterised elementary flows in this dataset.

## Usage Notes

The dataset supports planetary-boundary-based AESA of selected economic sectors across countries or Rest-of-World regions. We recommend using the open-source Python package, *PBAESA*^33^, to generate, import and use the dataset.

To perform a planetary-boundary-based AESA, the user must 1) quantify the environmental burdens of the selected economic sector across the planetary boundary categories via life cycle assessment, 2) calculate the sector-specific allocated share of the global safe operating space for each planetary boundary category, and 3) compare the environmental burdens against the allocated share of safe operating space to assess whether the sector is environmentally sustainable^3^.

To perform a life cycle assessment, the user has to apply the characterisation factors provided here to the life cycle inventory of the selected sector. The dataset is tailored to life cycle inventories derived from the standard and prospective ecoinvent v3.10.1 databases^13,19^. Additionally, the user can import all characterisation factors automatically in the Brightway framework^34^ via create_pbaesa_methods in *PBAESA*. The data can be adapted for other life cycle inventory databases and life cycle assessment software. In addition to process-based LCA, the data can also be applied in environmentally extended input–output analysis studies.

To determine the allocated share of safe operating space, the user must multiply the global safe operating space (see the second sheet *Characterization factors_for_eco3101.xslx*) with the sector-specific allocation factors. The dataset provides allocation factors for the year 2022 that are calculated by using four allocation principles that combine population-based allocation with economic value-based allocation, considering direct or total, and consumption- or production-based value generated by the economic sector.

We recommend comparing against shares of safe operating space determined by using the allocation factors based on both total consumption- and production-based value, as each factor captures a distinct value perspective. Using total value rather than direct value is preferable to ensure consistency with the life cycle approach used to determine environmental burdens. In cases where the environmental burdens only cover burdens caused directly by the economic sector, i.e., gate-to-gate burdens, allocation factors based on direct consumption- and production-based value should be used.

When selecting allocation factors, the user must ensure that the included processes, the functional unit, and the temporal and geographic scope of the sector for which the allocation factor is defined are consistent with those of the life cycle assessment. For example, if the environmental burdens are calculated for electricity generated in Germany from wind and solar technologies to meet German electricity demand in 2050, the allocation factors must be derived from economic value data representing the same electricity production processes (wind and solar), and covering the same functional unit (German electricity demand), geographical scope (Germany) and temporal scope (2050).

If the processes included, the functional unit, or the geographic scope of the anthropogenic system under assessment do not match those of the economic sector for which allocation factors are available, the user may aggregate or disaggregate economic sectors accordingly. When aggregating sectors, however, double counting may arise (cf. Section 2.1.1). Any aggregation procedure and the associated risk of double counting should therefore be documented transparently. The dataset does not provide allocation factors for disaggregating economic sectors. We recommend performing such disaggregation based on the share of the sector’s economic value attributable to the assessed anthropogenic system, for example using turnover data. If the temporal scope of the life cycle assessment does not match that of the available allocation factors, different approaches are recommended depending on the assessment year. For life cycle assessments with a temporal scope of 2022 or later, we recommend using the allocation factors included in the dataset. For earlier years, we recommend computing historical allocation factors using get_all_allocation_factors in *PBAESA*. The provided allocation factors, as well as the functions used to compute historical allocation factors, are based on EXIOBASE and comply with the EXIOBASE license terms.

## Data Availability

The allocation factors are openly accessible in the Zenodo repository *Allocation Factors for Absolute* *Environmental Sustainability Assessment*^30^ in the Excel file *Allocation Factors.xlsx*. The characterisation factors are openly accessible in the Zenodo repository Characterisation *Factors for Planetary-Boundary-Based Absolute Environmental Sustainability Assessment*^32^ in the Excel file *Characterisation factors_for_ecoinvent3101.xslx*.

## Code availability

All custom code is openly accessible via GitHub and preserved in in the Zenodo repository *PBAESA: A Python Package for Planetary-Boundary-Based Absolute Environmental Sustainability Assessment*^33^. We provide a dedicated Python package, *PBAESA*, which can be installed through *pip* and enables users to automatically generate, import, and use the dataset.
